# First case of *Kluyveromyces marxianus* (*Candida kefyr*) late onset keratitis after lamellar endothelial corneal graft

**DOI:** 10.1016/j.mmcr.2021.02.001

**Published:** 2021-02-12

**Authors:** Alexander M. Aldejohann, Johanna Theuersbacher, Lukas Haug, Olga S. Lamm, Grit Walther, Oliver Kurzai, Jost Hillenkamp, Daniel Kampik

**Affiliations:** a Julius-Maximilians-University, Institute for Hygiene and Microbiology, Josef-Schneider- Str.2, 97080, Würzburg, Germany; b University Hospital of Würzburg, Department of Ophthalmology, Josef- Schneider-Str. 11, 97080, Würzburg, Germany; c Julius-Maximilians-University, Institute of Pathology, Josef-Schneider-Str. 2, 97080, Würzburg, Germany; d National Reference Center for Invasive Fungal Infections NRZMyk, Leibniz Institute for Natural Product Research and Infection Biology - Hans Knöll Institute (HKI), Adolf-Reichwein-Str. 23, 07745, Jena, Germany

**Keywords:** Fungal keratits, *Kluyveromyces marxianus* (*Candida kefyr*), DMEK, Corneal transplantation, Voriconazole, Amphotericin B

## Abstract

We present a case of *Kluyveromyces marxianus* keratitis nine months after Descement's membrane endothelial keratoplasty (DMEK) in a patient with Fuchs endothelial disease. Endothelial scraping revealed this rare yeast infection at the interface between graft and host cornea. Immediate antifungal treatment with intracameral and corneal intrastromal injections of voriconazole and amphotericin B remained unsuccessful, requiring penetrating keratoplasty. This case highlights the challenging management of keratomycosis in patients with endothelial grafts.

## 1 Introduction

Keratomycosis is a severe sight-threatening eye infection, conferred by both *Candida* *species* and filamentous fungi, which imposes a significant health issue of growing global concern [[1], [2], [3], [4], [5], [6]]. While keratomycosis is a relatively common event in tropical and subtropical regions, the incidence of fungal keratitis remains rare in moderate climate countries within Europe [2,5,6]. However, between 2003 and 2017 a 5-fold increase has been observed in different European countries, from 0.32 to 1.53 cases per million inhabitants per year [3,4].

Etiologically, “independent” (i.e. social, economic, regional) and personal risk factors including corneal trauma, usage of contact lenses, prolonged usage of steroid eyedrops, ocular surface diseases and previous corneal transplants can be differentiated [[1], [2], [3]]. Though contaminations of transplants with *Candida* *species* leading to keratitis or endophthalmitis after partial or full keratoplasty are rare events, differences in tissue preparation and storage standards of corneal transplants might serve as a possible explanation. Furthermore, the fortification of storage media with antifungal substances is handled divergently in eye banks across Europe and the US [7].

The transplantation of a lamellar endothelial corneal graft (DMEK, *descemet membrane endothelial keratoplasty)* is a less invasive and less immunogenic standard technique in comparison to full thickness keratoplasty [8]. The diseased corneal host tissue is exchanged by a donor lamellar graft comprising the basal lamina with the endothelial cell monolayer. Yeast infections after graft insertion have been reported [7,9]. Especially in treatment of Fuchs endothelial dystrophy DMEK has increasingly evolved to a standard procedure due to good long-term efficacy and fast increase of visual acuity [10,11]. Fuchs dystrophy is characterized by bilateral loss of corneal endothelial cells causing vision loss due to corneal edema. If medical treatment is unsuccessful, corneal transplantation is required.

The dairy yeast and facultative opportunistic fungal pathogen *Kluyveromyces marxianus* (syn.: *Candida kefyr*) -formerly *Candida pseudotropicalis*- is rarely associated with human infection and scarcely described as transient eye flora [12,13]. Here we present a case of keratomycosis caused by *Kluyveromyces marxianus* after DMEK.

## 2 Case presentation

A 69-year-old male patient suffering from Fuchs endothelial dystrophy predominantly affecting the right eye was admitted to the ophthalmology department for elective DMEK, which was performed without any complications (day 0). Notably, 13 years earlier, the patient had undergone cataract surgery with insertion of an artificial lens as well as a trabeculectomy for pseudoexfoliation (PEX) glaucoma. In PEX glaucoma, metabolism of the extracellular matrix is disturbed. The deposition of fibrillary material causes instability of the lens zonula and an increase in intraocular pressure.

After DMEK surgery, dexamethasone eye drops (1mg/ml) were applied six times a day, tapered weekly till day 42, to prevent rejection and to prolong graft survival. The cornea cleared and visual acuity increased from 0.15 (20/125) to 0.5 (20/25) within the following three weeks.

After a symptom-free period of three months, a PEX-related luxation of the artificial lens occurred requiring lens exchange with implantation of an iris-fixated, retropupillar intraocular lens (day 104). The cornea remained clear postoperatively, visual acuity was 0.5 (20/25).

Almost nine months after DMEK (day 264), sudden pain of the right eye set in. Redness and corneal edema were initially interpreted as graft rejection and steroid eye drops were administered (day 279–285: prednisolone acetate 10 mg/ml four times daily; later changed to preservative-free dexamethasone eye drops 1 mg/ml three times daily, day 286–288).

**Fig. 1:**
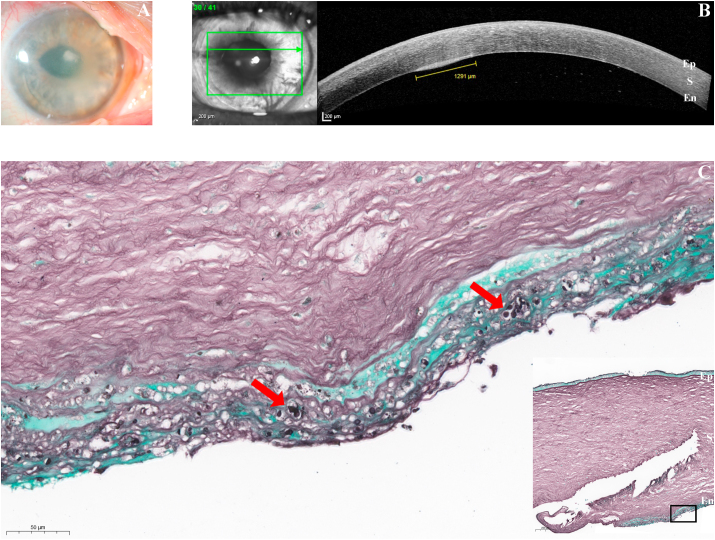
Late onset *Kluyveromyces marxianus* keratitis after lamellar corneal transplantation (DMEK, descemet membrane endothelial keratoplasty). A. Clinical findings nearly 9 months (288 days) after DMEK surgery: Paracentral corneal infiltrate with surrounding corneal opacity due to epithelial and stromal edema. B. Cross-section of the cornea by in vivo optical coherence tomography reveals localization of the infiltrate (1291 μm in diameter) between graft lamella and host cornea (Ep: epithelium, S: stroma, En: endothelial transplant lamella). C. Fungal structures (red arrows) in Grocott stain with surrounding mild inflammation. (Ep: epithelium, S: stroma, En: endothelial transplant lamella). (For interpretation of the references to colour in this figure legend, the reader is referred to the Web version of this article.)

As vision deteriorated further the patient was referred to our ophthalmology department on day 288. A dense whitish paracentral corneal infiltrate became evident, accompanied by an entirely opaque cornea due to stromal edema (Fig. 1A). Optical coherence tomography revealed a hyperdensity of 1.3 mm in diameter localized between graft and host cornea (Fig. 1B).

Based on these findings we decided to broaden the diagnostic approach by tapping the anterior chamber and acquire an endothelial scraping in the area of the DMEK lamella harbouring the suspicious infiltrate. Local therapy consisted of an hourly alternating antibiotic regimen (day 288–291: ofloxacin 3 mg/ml and gentamicin sulphate 5 mg/ml).

While initial Gram staining of the scraping material showed no evidence of microbial pathogens, typical small yeast-like white colonies were observed both on chromogenic media (CHROMagar, Becton Dickinson, USA), blood and chocolate agar after 24 hours of incubation at 36 °C. The species was then identified as *Kluyveromyces marxianus* by Matrix-Assisted Laser Desorption/Ionisation-Time of Flight (MALDI-TOF) mass spectrometry (MS) (Biomerieux Paris, France, VITEK MS database version 3.2) and finally confirmed via sequencing the internal transcribed spacer of the ribosomal DNA (ITS-rDNA). The ITS sequence of the isolate showed 100% identity with that of the ex-type strain of *Kluyveromyces marxianus* CBS 712 (GenBank accession number NR_111,251). The strain is deposited in the Jena Microbial Resource Collection (JMRC:NRZ:2921).

Immediate susceptibility testing was performed by VITEK 2 automated system (Biomerieux, Paris, France) using an AST-YS08 testing cartridge (acc. to CLSI). Additionally, Sensititre Yeast One YO10 microbroth dilution (acc. to CLSI) and a EUCAST broth microdilution were conducted (Table 1).

**Table 1:** *Kluyveromyces marxianus* (syn.: *Candida kefyr*) susceptibility testing results after 24h. Antifungal agents: anidulafungin (AND), amphotericin B (AMB), micafungin (MIC), caspofungin (CAS), 5-flucytosine (5-FC), posaconazole (POS), voriconazole (VOR), itraconazole (IT), fluconazole (FLU).

Testing method	Acc. to reference method	Minimal inhibitory concentrations (MICs) of antifungal agent (μg/ml)								
		AND	AMB	MIC	CAS	5-FC	POS	VOR	IT	FLU
VITEK 2	CLSI	–	0.5	0.12	–	–	–	≤0.12	–	1
YO	CLSI	0.12	1	0.12	0.12	≤0.06	0.12	≤0.06	0.12	0.5
Broth Microdilution	EUCAST	0.03	0.125	–	–	–	≤0.016	≤0.016	≤0.016	0.5

A combination therapy of oral fluconazole (day 291 onwards: 400 mg daily loading dose; 200 mg daily maintenance dose) and hourly eye drops with voriconazole (1%) were begun followed by regular voriconazole (2%) injections. A total of four voriconazole injections were administered intracamerally, intrastromally and subconjunctivally (day 292, 294, 299, 301).

Unfortunately, the infiltrate remained refractory to this treatment, even after regimen was switched to amphotericin B (50 μg/ml in 0.1ml) injections (day 305). On day 306, emergency full thickness keratoplasty had to be performed, again followed by intracameral application of amphotericin B.

The explanted graft revealed a slightly discontinuous Descemet membrane, whereas the multilayered corneal epithelium showed no significant abnormalities. Histopathology confirmed *Candida* infection. Fungal structures (red arrows) were identified in both PAS and Grocott stain with surrounding mild inflammation (Fig. 1C).

After keratoplasty, the new graft showed no evidence of recurrence. As prophylaxis, antifungal therapy was continued with hourly application of topical voriconazole (1%) and two intracameral injections of amphotericin B (50 μg/ml in 0.1ml, day 319 and 322). For the remaining follow-up period up to day 354, findings were stable without signs of fungal activity, so that antimycotic topical and systemic therapy was discontinued.

## 3 Discussion

Yeast infection is a rare complication of DMEK, occurring in 0.15% of the cases [14]. In the majority, causative pathogens can be obtained from direct biopsy. However, recent studies indicate that *Candida*-positive cultures of the corneal donor rim (tissue left over from transplantation) may be a valuable parameter in predicting fungal keratitis prior to the onset of clinical symptoms [7]. However, our patient developed no evidence of infection even four weeks after surgery, and neither did the donor rim show any signs of infection and was therefore discarded. This procedure is supported by the fact that longer preservation time is not necessarily correlated with higher incidences of donor rim contamination. In fact, only a small fraction of cases with positive donor rim cultures develop post-keratoplasty infection [15]. Therefore, it remains debatable whether the patient would have benefited from a prolonged donor cornea conservation.

*Kluyveromyces marxianus* is a rare but severe and life-threatening cause of bloodstream infection, which is underlined by the fact that *Kluyveromyces marxianus* candidiasis is associated with a higher mortality in ICU patients compared to *C. albicans* [16]. Hematologic malignancies are considered as a risk factor. Interestingly, a seasonal peak of colonisation and infection is observed during summertime, which might be related to a lack of refrigeration in dairy products [13].

In our case the patient developed first symptoms of keratitis nine months after DMEK. Fontana et al. described onset of bacterial or fungal keratitis as late as four months after surgery [17]. The special localization of the yeast sequestered between graft and host cornea could influence the late onset of infection and makes it less accessible by topical or systemic therapy [7,9]. However, therapeutic approaches do exist combining systemic therapy with multiple intrastromal and intracameral antimycotic injections, avoiding full thickness keratoplasty in *Candida* lamellar infection [9].

Despite some *Kluyveromyces marxianus* in vitro data, no official breakpoints are established due to the paucity of clinical, pharmaceutical and epidemiological information [18]. Only EUCAST provides one fluconazole non-species related breakpoint for *Candida* based upon PK/PD data, which is defined as susceptible ≤2 μg/ml.

Our antifungal regimen included oral administration of fluconazole accompanied by intracameral and intrastromal injections of voriconazole, which was considered effective -at least in vitro- according to the susceptibility data (Table 1).

Previous case reports clearly indicate that intracorneal injections of voriconazole are useful alternatives challenging deep stromal *Candida* infections, especially when full thickness keratoplasty is clinically thought to be too invasive at that particular timepoint [19]. Voriconazole is reported effective against different *Candida species* in-vitro, but overall pharmacokinetic and pharmacodynamic data is limited. Even so, experimental studies suggest a demand of repeated drug administration at the site of infection, due the risk of accelerated loss of concentration below MIC of common fungi, which we met with multiple intracarmal and intrastromal injections [20]. Fluconazole was given as an attempt to further strengthen local azole concentrations, being aware that corneal drug penetrations of systemically added antifungals are usually poor.

Notably, comparing both EUCAST and CLSI methodologies performed on different testing devices (in-house broth microdilution versus semiautomatic- and automatic commercial broth microdilution), obtained minimum inhibitory concentrations (MICs) are in a similar range.

A change to amphotericin B was considered as a last attempt for medical treatment. It is debatable whether this change should have been considered earlier or even in first place, but it illustrates the dilemma of susceptibility testing in vitro and the concordant effect in vivo at the site of infection.

Nonetheless, graft removal was inevitable, underlining the complexity of treating this rare infection by constantly re-balancing the value of antifungal therapy with the lack of in vivo breakpoints against surgical measures, which will ultimately remove the infected focus but result in a new full thickness graft with a risk of reinfection and other complications.

Further interdisciplinary clinical studies and case reports are warranted not only to elucidate the role of *Kluyveromyces marxianus* as a rare pathogen but also to develop new treatment strategies for this challenging infection.

## Funding source

The German National Reference Center NRZMyk is funded by the Robert Koch Institute from funds provided by the 10.13039/501100003107German Ministry of Health (grant no. 1369-240).

## Consent

Written informed consent was obtained from the patient or legal guardian(s) for publication of this case report and accompanying images. A copy of the written consent is available for review by the Editor-in-Chief of this journal on request.

## Declaration of competing interest

There are none.
